# Supplementary material for: Complexome profiling of the Chlamydomonas psb28 mutant reveals TEF5 as an early PSII assembly factor
Source: Plant Cell. 2025 Mar 17;37(6):koaf055. doi: 10.1093/plcell/koaf055 (PMC12164590; doi:10.1093/plcell/koaf055)
Supplement: koaf055_Supplementary_Data [file koaf055_supplementary_data.zip › psb28_interactive.zip!/plots/intensity/qualityPlots/peptides/FSD1_Cre10.g436050.t1.2.html]

### Cre10.g436050.t1.2

Trivial name: FSD1  
SeqCoverage [median]: 14.10  
Mass: 25.91 kDa  
  
 

| Sequence | MissedCleavage | Mass [Da] | wtIntensity | muIntensity | Score |
| --- | --- | --- | --- | --- | --- |
| LINWDAVAQR | 0 | 1184.63 | 0.00 | 559.82 | 87.48 |
| RPDYITTFMEK | 1 | 1399.68 | 0.00 | 600.20 | 45.26 |
| SPNAVNPVVEGK | 0 | 1209.64 | 264.16 | 4141.96 | 126.19 |
|  |  |  | 264.2 | 5302.0 |  |
